# X-band MMICs for a Low-Cost Radar Transmit/Receive Module in 250 nm GaN HEMT Technology

**DOI:** 10.3390/s23104840

**Published:** 2023-05-17

**Authors:** Hyeonseok Lee, Hyeong-Geun Park, Van-Du Le, Van-Phu Nguyen, Jeong-Moon Song, Bok-Hyung Lee, Jung-Dong Park

**Affiliations:** 1 Division of Electronics and Electrical Engineering, Dongguk University, Seoul 04620, Republic of Korea; 2 Yongin Research Institute, Hanwha Systems, Yongin-si 17121, Republic of Korea

**Keywords:** gallium nitride (GaN), transceiver, high-power amplifier (HPA), low-noise amplifier (LNA), driving amplifier (DA), T/R switch

## Abstract

This paper describes Monolithic Microwave Integrated Circuits (MMICs) for an X-band radar transceiver front-end implemented in 0.25 μm GaN High Electron Mobility Transistor (HEMT) technology. Two versions of single pole double throw (SPDT) T/R switches are introduced to realize a fully GaN-based transmit/receive module (TRM), each of which achieves an insertion loss of 1.21 dB and 0.66 dB at 9 GHz, IP_1dB_ higher than 46.3 dBm and 44.7 dBm, respectively. Therefore, it can substitute a lossy circulator and limiter used for a conventional GaAs receiver. A driving amplifier (DA), a high-power amplifier (HPA), and a robust low-noise amplifier (LNA) are also designed and verified for a low-cost X-band transmit-receive module (TRM). For the transmitting path, the implemented DA achieves a saturated output power (*P*_sat_) of 38.0 dBm and output 1-dB compression (OP_1dB_) of 25.84 dBm. The HPA reaches a *P*_sat_ of 43.0 dBm and power-added efficiency (PAE) of 35.6%. For the receiving path, the fabricated LNA measures a small-signal gain of 34.9 dB and a noise figure of 2.56 dB, and it can endure higher than 38 dBm input power in the measurement. The presented GaN MMICs can be useful in implementing a cost-effective TRM for Active Electronically Scanned Array (AESA) radar systems at X-band.

## 1. Introduction

The recent remarkable development of solid-state semiconductors has enabled a high-resolution remote sensor to be implemented. In particular, the transition from classical magnetron radar to solid-state semiconductor radar made the coherence characteristics available and various signal processing techniques possible. This method can realize technologies such as Doppler processing, pulse compression, and beamforming used in modern radars. Particularly, an Active Electronically Scanned Array (AESA) radar utilizing beamforming technology enables accurate ranging and detection by adjusting the direction of the shared antenna beam through electrical control of phase and size. AESA radars have recently been widely used in various sectors of military aviation, surveillance, and civil applications. AESA radar systems rely heavily on the performance of the Transmit (Tx)/Receive (Rx) module (TRM) for each antenna element since it is a crucial module in determining the SNR of reflected signals and its cost. Thousands of TRMs are integrated depending on the applications [1]. Hence, it is essential to implement a compact and cost-effective TRM for a competitive AESA radar system for various applications.

**Figure 1:**
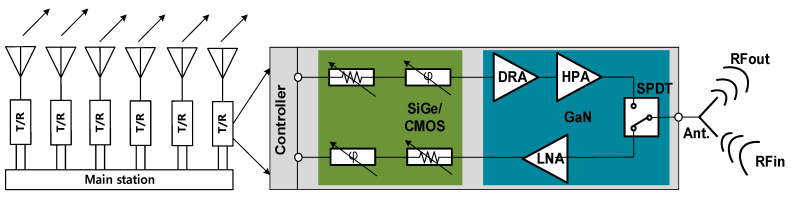
Transceiver module in an AESA radar system.

Figure 1 depicts transceiver module system including a TRM block in an AESA radar system. The TRM front-end block consists of a High Power Amplifier (HPA) with a Driving Amplifier (DA) at the Tx chain, a Low Noise Amplifier (LNA) at the Rx chain, and a Single-Pole Double-Throw (SPDT) Transmit/Receive (T/R) switch controlled by the transmit and receive functions.

Traditionally, TRM front-end blocks have been implemented with GaAs technologies considering the superb device performance at microwave and millimeter-wave regimes. Recently, research on GaN technologies has been widely conducted across the semiconductor sectors, including research on efficient full-spectrum WLEDs utilizing bandgap engineering [2] and GaN radiation hardness for space-applied electronics [3]. In particular, research on GaN-based RF front-end mainly relies on the large bandgap characteristics. By replacing TRM front-end blocks with GaN technology, it is possible to implement a low-cost and compact TRM by eliminating a bulky ferrite circulator and external limiters in front of the GaAs LNA. The high breakdown voltage of the GaN High Electron Mobility Transistor (HEMT) enables a high voltage swing of the HPA with a higher supply voltage, resulting in a higher power density than GaAs HEMTs [4]. This enables the use of smaller device sizes and less complex output matching networks, which may result in more compact and more power-efficient blocks in the GaN technology than GaAs counterparts.

The design of RF circuits utilizing GaN HEMT devices with excellent characteristics requires accurate GaN HEMT device modeling studies. Early empirical models were proposed by Angelov et al. [5] and Sadi et al. [6]. Later, based on the device physics of the model, the ASM-HEMT model [7], MVSG HEMT model [8], and EPFL HEMT model were proposed for improved GaN HEMT modeling [9]. During the last decade, GaN HEMT device modeling studies have enabled foundries to provide commercially viable GaN HEMT design services, such as modeling the NQS effect [10], improving HEMT models at cryogenic temperatures [11], and modeling the charge trapping effect [12] to improve the modeling of nonlinear characteristics of HEMT devices.

For the LNA in the Rx path of a conventional TRM, GaAs HEMT has been widely employed due to its high transconductance and superior noise performance. However, considering feasible high input power interferences, external limiters are necessary to protect the GaAs HEMT at the first stage of the LNA, which compromises the noise performance and increases the manufacturing cost. Moreover, a conventional Rx implemented with GaAs MMICs requires the use of bulky ferrite circulators to isolate the Tx and Rx paths.

The high-power handling capability of the GaN HEMT allows a SPDT switch to operate as a compact Tx/Rx duplexer. Since the SPDT switch is located at the input port of the antenna element, its performance significantly affects on the overall TRM block. In order to achieve the required SNR of the entire Rx path, it is essential to minimize the insertion loss (*IL_SPDT_*) of the SPDT switch as it degrades the SNR by twice the *IL_SPDT_*. By substituting a bulky ferrite circulator and the input limiter blocks with a compact GaN T/R switch, the performance of the GaN Rx chain can be comparable with the GaAs Rx chain with a lower manufacturing cost and more compact module size.

In this paper, core MMIC blocks for a cost-effective TRM aiming at the X-band AESA radar system are presented. All the core blocks such as the low insertion loss SPDT switch, and low-noise LNA for Rx chain, DA, and HPA were implemented in 250 nm GaN on the SiC HEMT process. For SPDTs, the impact of the presence or absence of a compensation inductor on the overall value of insertion loss and bandwidth is discussed. The HPA design introduces a pulse-induced test to avoid stability issues. In the design of LNA, it is demonstrated that utilizing a high gate resistor and a drain current limiter ensures high robustness. A comparison table is provided to compare the performance of each block with other recently reported GaN-based works in the X-band, which demonstrate the feasibility of the fully GaN-based TRM.

## 2. X-band Transceiver Front-End Design

In this work, all the GaN MMICs were realized in 0.25 µm GaN/SiC HEMT power device technology. The GaN heterostructure is grown on a 100 µm SiC substrate by plasma-enhanced chemical vapor deposition (PECVD). In this process, two metal layers can be routed: MET1 of 1.1 µm thickness, and MET2 of 4 µm thickness, which can be connected using a via-layer of 0.27 µm thickness. The cut-off frequency *f_T_* and the maximum frequency of oscillation *f_max_* are 25 GHz and 82 GHz, respectively.

### 2.1. SPDT Switch as Duplexer

Working as a duplexer, the GaN SPDT switch is placed right after each antenna element and connects both the receive and transmit paths. Thus, it requires an extremely low insertion loss and high isolation between Tx and Rx ports. Exhibiting a low insertion loss may improve the overall SNR of the implemented TRM, and the high isolation minimizes high power signal leakage from the Tx to Rx path in transmit mode that can permanently damage the gate diode of a LNA.

**Figure 2:**
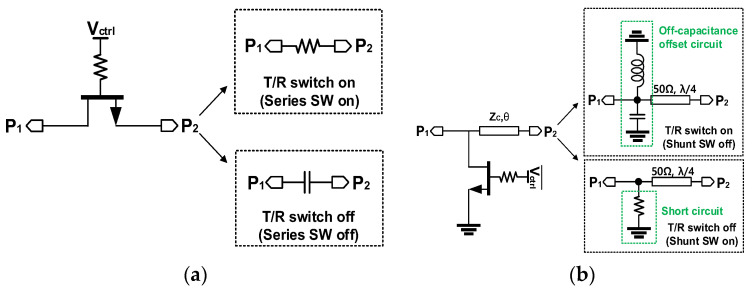
Switch mode of (**a**) series FET switch and (**b**) parallel FET switch.

Figure 2 presents two types of FET switches, which are series switches and parallel switches, and both are used to construct a conventional single pole double throw (SPDT). Each switch in the SPDT turns on and off inversely depending on the gate control voltage (V_H_ = 0 V, V_L_ = −26 V). Notably, the on-state power is limited by the maximum channel current (*I_MAX_*) of the series switch of which the typical value at *V_GS_* = 0 V is about 1 A/mm. Thus, the maximum available power through the series HEMT is approximately 25 W for a HEMT with a gate width of 1 mm. Thus, it can be a threat of using a SPDT switch as a duplexer for high-power applications (typically 25 W for recent designs). Moreover, the series HEMT switch induces a relatively large insertion loss since it is in the main path of signal conduction. It is noted that the maximum power of the off-state switch should consider the breakdown voltage (*V_BD_*), the gate control voltage (*V_ctrl_*)*,* and the pinch-off voltage (*V_p_*). To maximize the voltage swing, *V_ctrl_* is selected to be centered between *V_BD_* and *V_p_* [13]. Owing to the high band gap property of GaN, the breakdown voltage (*V_BD_*) is 120 V under a drain current density of 1 mA/mm in this GaN process. With a −2.6 V pinch-off voltage, an off-state power can be handled up to a hundred watts. Since the reliability of the parallel switch is mainly dependent on the off-state power handling rather than on-state power handling, the SPDT can achieve a low insertion loss with high output handling capability when only parallel HEMT switches are used with λ/4 transmission-lines (T-lines) for the isolation.

**Figure 3:**
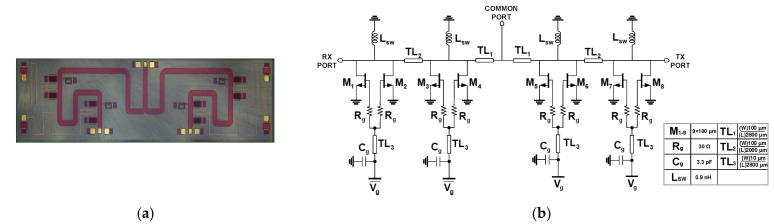
(**a**) Chip photo and (**b**) schematic of the SPDT T/R switch version-A (SW-A).

In this work, we implemented two types of SPDT T/R switches. The first T/R switch (SW-A) employs shunt inductors to compensate parasitic capacitance of shunt transistors to improve isolation and bandwidth. The second T/R switch (SW-B) is more focused on the low insertion-loss, which excludes the shunt inductors. SW-A is advantageous in achieving a wider bandwidth with improved isolation owing to the resonant inductors. However, the additional lossy inductors degrade the overall insertion loss of the switch. Figure 3 depicts the layout and the schematic of SPDT T/R switch version-A (SW-A). The designed T/R switch occupies a chip size of 5.7 mm × 1.5 mm. The proposed T/R switch has a symmetric structure, and each arm consists of four parallel HEMTs with transmission lines without any series HEMT switches. The simulations have been conducted in Keysight ADS, and all the routing lines in the design were optimized with the Momentum EM simulator in ADS. The size of all shunt transistors is optimized to achieve the minimum insertion loss while providing good isolation and linearity. When the Tx path is turned on, that means M_1–4_ should be turned on to make a short circuit, which is consequently seen as open by the λ/4 transmission line, TL_1_. Thus, M_1–8_ need to be large enough to minimize the on-state resistance. Considering these aspects, the width of M_1–8_ is 9 × 100 μm.

Since all the active devices are depletion HEMT, applying 0 V control voltage is enough to keep the transistor at the on-state and can also provide low resistance for short circuits. When the Tx path is turned off, an inserted shunt inductors resonate with the parasitic capacitance of the off-state transistors, which decreases the loading of the shunt transistor remarkably. Since this design should be integrated with other blocks (e.g., LNA, PA) to construct a TRM utilizing 26 V for the HPA and DA, *V_ctrl_* = −26 V is chosen. It is noteworthy that the used T-lines need to be wide enough to carry the input power level as expected from the output of HPA. The characteristic impedance is calculated and tuned deliberately with the Momentum EM simulation in ADS. In previous works, several k-Ohm resistors were used to decouple the switch circuit from the common gate ground [13,14,15,16,17,18,19]. However, the gate leakage current combined with a high-value resistor induces a distinctive change to the effective control voltage, which can result in a huge degradation in the overall performance of the switch, specifically for the on-state HEMT as it can change state from on- to off-state. Hence, in our work, a λ/4 T-line with a high characteristic impedance (narrow width) is used at the gate control line for the improved isolation purpose. In addition, a 30 Ω resistor is inserted between the gate and the control line to enhance stability.

**Figure 4:**
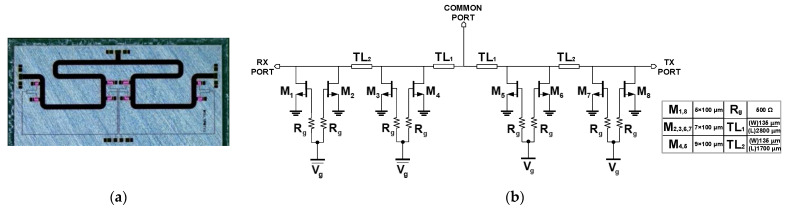
(**a**) Chip photo and (**b**) schematic of the SPDT switch version-B (SW-B).

The chip photo and the schematic of SW-B are presented in Figure 4. Different from SW-A, SW-B removes the parallel inductors to improve the overall insertion loss. The designed SW-B occupies a chip size of 5.3 mm × 2.6 mm. To reduce the parasitic capacitance, the widths of M_1,8_ and M_2,3,6,7_ are changed to 5 × 100 μm and 7 × 100 μm, respectively. Meanwhile, the width of M_4,5_ remained at 9 × 100 μm to guarantee the small channel resistance with a T/R switch off-state. Different from SW-A, a gate resistor R_g_ = 500 Ω was adopted for the isolation purpose, and the degradation due to the voltage drop was not measured as described the Section 3.1.

### 2.2. High Power Amplifier

**Figure 5:**
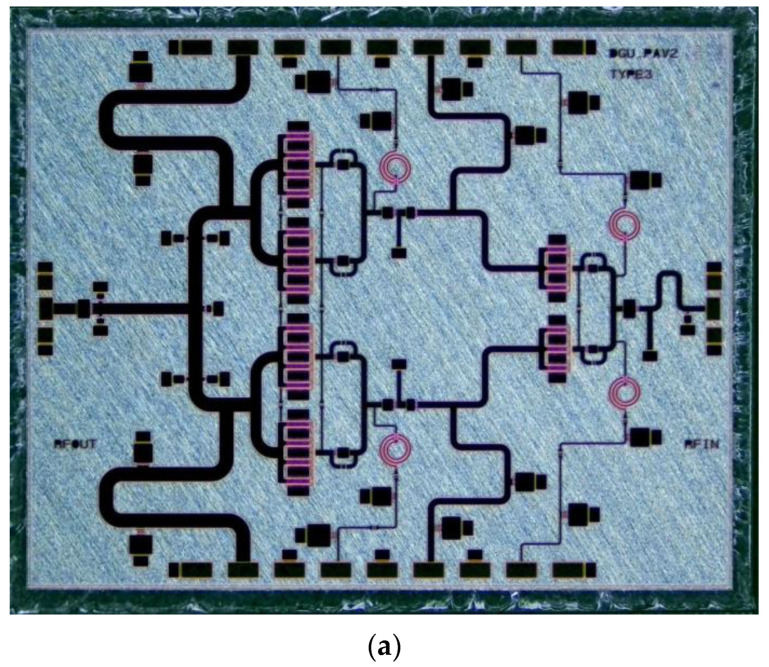

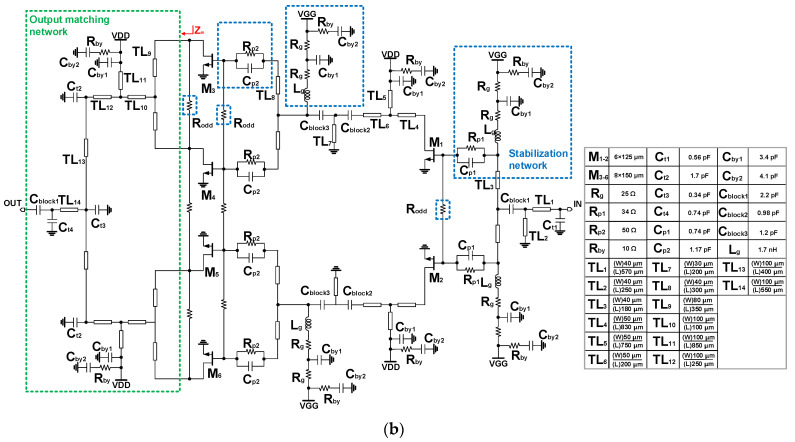
(**a**) Chip photo and (**b**) schematic of the high-power amplifier (HPA).

The designed High Power Amplifier (HPA) architecture utilizes a four-way power combining structure at the power stage with a cascaded two-stage configuration. Figure 5a shows the photo of the implemented HPA, which occupies 4.6 mm × 3.7 mm. Figure 5b presents the schematic of the HPA. In this HPA design, individual source via (ISV)-type HEMTs are utilized to minimize the source degeneration from the via to the ground.

The HEMT at the first stage (M_1–2_) and the final stage (M_3–6_) has a gate periphery ratio of 1:3.2 with a width of 8 × 150 μm and 6 × 125 μm, respectively. A parallel RC network and a gate bias resistor are implemented in each gate of the transistor to enhance stability. Moreover, in-phase RF signals pass through each four path at the final stage. In this condition, an odd-mode oscillation may occur due to the electric asymmetry from the different DC supply paths of each transistor. To suppress feasible odd-mode oscillation, a resistor between transistors, R_o_ = 17 Ω is placed at the final stage. To avoid undesired oscillation from the power supply perturbation, the quality-factor (Q-factor) of the bypass capacitor network was degraded by adding a 10 Ω resistor in series with the bypass capacitor.

**Figure 6:**
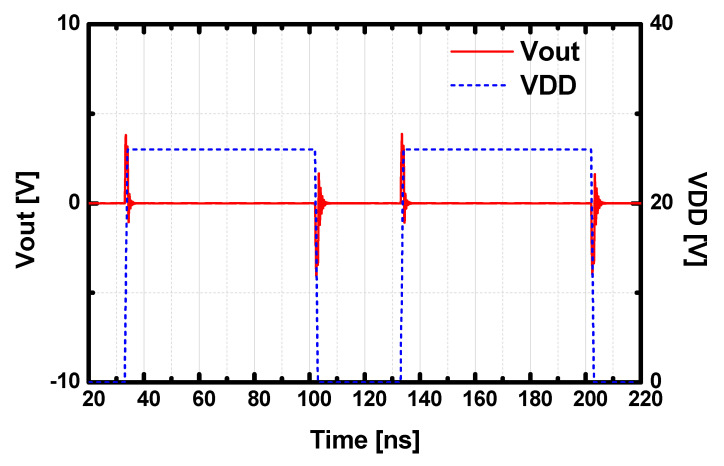
Simulated pulse-induced stability result in the time domain.

Theoretically, Rollet’s k-stability analysis can be usefully applied to check the unconditional stability of the two-port network at the small-signal regime. Unfortunately, a realized HPA cannot be properly modeled as a two-port network since it includes multiple biasing ports. Hence, oscillations may not be properly detected only with the k-stability test from the small-signal two-port S-parameters. To guarantee the stability of the designed HPA, we applied a pulse-induced stability test in the time domain to check undesirable oscillations from the power supply interferences. As presented in Figure 6, a periodic pulse perturbation was excited from the power supply, and the output voltage was investigated in the time domain. In the designed HPA architecture, the fluctuations were forced to be underdamped with various stabilization networks such as the odd-mode resistors, bypass capacitor networks with a low Q-factor, and parallel RC networks at the gate of each HEMT. The simulation shows that the output voltage was settled quickly under the strong perturbations from the supply.

**Figure 7:**
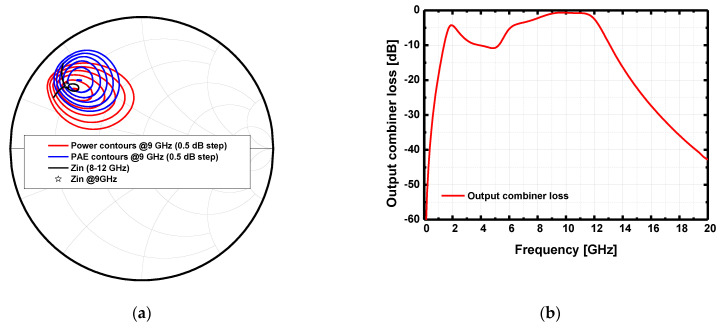
(**a**) Load-pull simulation result and (**b**) the loss of output combiner.

Figure 7a presents the load-pull simulation result plotted in the Smith chart, and the insertion loss of the output combiner is shown in Figure 7b. To suppress undesired out-of-band signals, the output-matching network was designed to achieve a bandpass filter property. The input impedance of the output combiner at 9 GHz is 10.8 + j19.43 Ω. The combiner loss at 9 GHz is 0.96 dB, which is an acceptable value for the four-way combiner, while the out-of-band suppression of the combiner is distinctive with a sharp skirt characteristic.

### 2.3. Driving Amplifier

**Figure 8:**
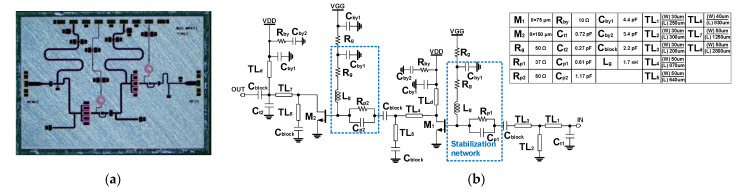
(**a**) Chip photo and (**b**) schematic of the driving amplifier (DA).

A two-stage driving amplifier (DA) with high power gain is used to drive the HPA at the saturation point with optimal peak power added efficiency (PAE). Figure 8 illustrates the chip photo and the schematic of the proposed amplifier. The size of the implemented DA is 4.4 mm × 2.8 mm. Since the designed power amplifier in Section 2.2 necessitates an input power of 32 dBm to achieve the saturation point, the DA should acquire an output power larger than 32 dBm.

The implemented DA was designed with ISV-type HEMTs to minimize the parasitic degeneration inductance from the via to the ground. To achieve a wide range of linear regions, the size of the device at the final stage (M_2_) was chosen to be 8 × 150 μm, which makes P_sat_ surpass 32 dBm. To keep the liner region from large input power and reduce the gain distortion within the operating power range, the size of the device at the final stage (M_1_) was selected to be 8 × 75 μm, resulting in the gate periphery ratio of 1:2. The input and output impedance was matched to 50 Ω considering the standalone measurement of each block and feasibility of the different compositions of MMIC blocks. The matching networks were comprised of capacitors and T-lines instead of inductors considering the reduction of the loss and modeling accuracy at the X-band.

**Figure 9:**
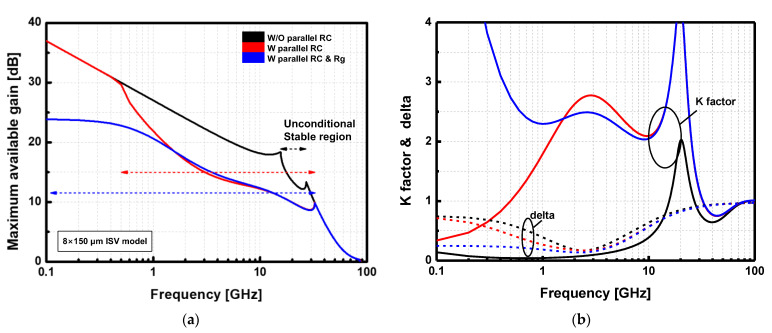
(**a**) Maximum available gain and (**b**) k factor (solid lines) and delta (dash lines) of stabilization cell with 8 × 150 μm device with different adoption of stabilization networks.

To increase the stability of the DA within the operating band, a parallel RC network was placed at the gate of each device. While the RC network enhances stability through overall frequency, the gain suppression was not enough to guarantee unconditional stability at a low-frequency regime. Thus, a series resistor was also placed in the path of the gate bias line of each device for improved stability at a low frequency. The values of resistance and capacitance of the parallel RC network were carefully chosen according to the size of each device while keeping unconditional stability with minimal gain degradation. Figure 9 shows a design example of a stabilization cell with an 8 × 150 μm ISV HEMT. By adding the parallel RC network with 50 Ω and 1.2 pF at the gate and a 50 Ω resistor in the gate bias line, the HEMT cell achieves unconditional stability from 100 MHz to 20 GHz range.

### 2.4. Low Noise Amplifier

**Figure 10:**
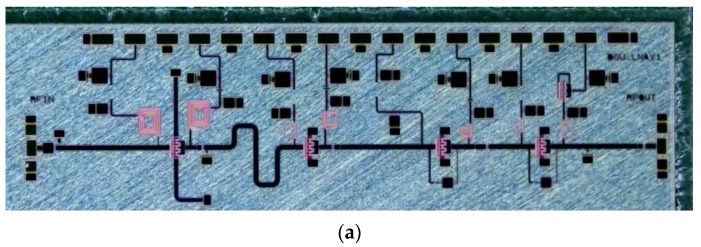

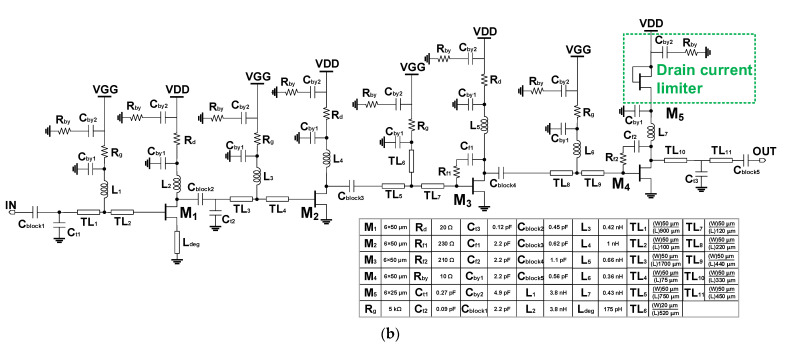
(**a**) Chip photo and (**b**) schematic of the proposed low noise amplifier (LNA).

The first stage was configured with an inductive source degeneration structure to achieve impedance and noise matching simultaneously. For the second stage, the RC-feedback is removed to improve the gain of the LNA, and the RC-feedback network was used for wideband operation with enhanced stability of the LNA in the last two stages. The optimal device size with a degeneration inductance was chosen to minimize the gain degradation for an improved noise performance of the overall receiver system with enhanced robustness from the high-power interference at the input of the LNA. While the smaller device has a low minimum noise figure (NF_min_), the larger device is advantageous for wideband matching. Figure 10 depicts the chip photo and the configuration of the proposed four-stage LNA with common source (CS) devices. In this simulation, a measurement-based noise model from the Foundry was used for the noise matching of the first stage of the LNA.

**Figure 11:**
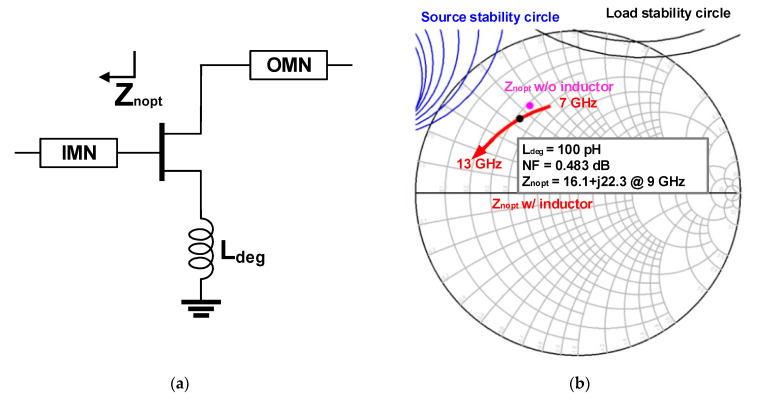

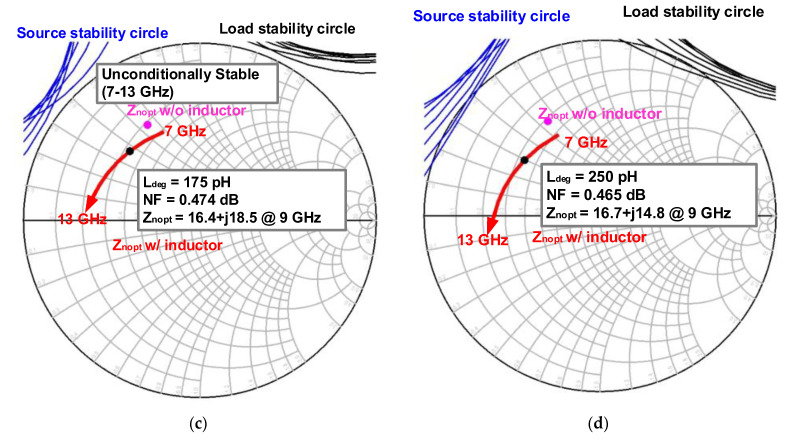
(**a**) Schematic of inductive degeneration and optimum noise figure impedance and stability check for 6 × 50 μm gate width with different degeneration inductance of (**b**) L_deg_ = 100 pH, (**c**) L_deg_ = 175 pH, and (**d**) L_deg_ = 250 pH.

The optimum noise impedance (Z_nopt_) of the selected device with 6 × 50 μm gate width is simulated as shown in Figure 11a. As illustrated in Figure 11b through Figure 11d, the degeneration inductance should be carefully selected to guarantee the unconditional stability that was achieved with L_deg_ = 175 pH.

**Figure 12:**
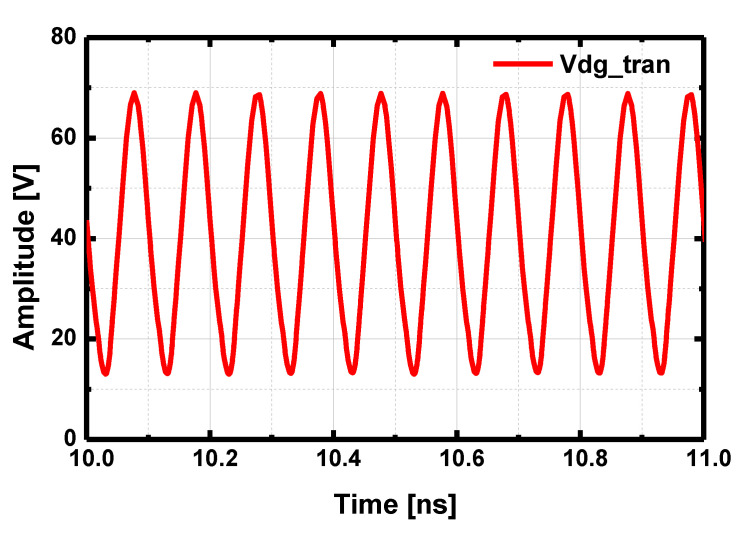
Transient simulation of the drain-gate voltage of the first stage HEMT in the LNA with Pin = 38 dBm.

For a GaAs LNA, the first stage of the LNA can be easily damaged from excessive RF input power, which necessitates a limiter for a robust Rx chain. To implement a GaN LNA without any input protection circuit, the designed LNA introduced a high gate resistor in the path of the gate bias line to reduce the gate leakage current from the high voltage swing at the gate by effectively lowering the gate bias voltage from the voltage drop of the gate resistor from the leakage current [20]. Figure 12 presents the drain-gate voltage of the first stage HEMT with P_in_ = 38 dBm (100 μs of pulse width and 10% of duty-cycle). The breakdown voltage of the gate-drain junction (V_BDG_) is 120 V in the PDK.

Different from the GaN LNA, a succeeding CMOS multi-function chip (MFC) can be seriously damaged even with 1 W power, which necessitates limiting the output power level of the LNA. By reducing the size of the final stage transistor, the control output power seems to be easy; however, a decrease in the size is limited to the specific design rule and aggravates the output impedance matching transformation ratio, which leads to the reduction of the bandwidth. To control the output power of the LNA and protect a succeeding silicon-based block, a diode-connected load was used at the final stage as shown in Figure 10b. The size of the limiter was carefully chosen considering the trade-off between the small signal gain reduction and the output power level control of the final stage. The output power of the LNA with the drain limiter was alleviated to 24 dBm.

## 3. Measurement Results

### 3.1. SPDT T/R Switches

**Figure 13:**
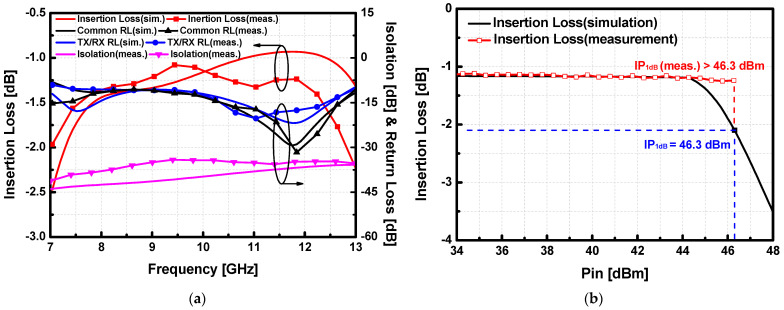
Simulated and measured (**a**) small-signal performance and (**b**) large-signal performance at 9 GHz results of the proposed SPDT switch version-A (SW-A) under RF pulsed conditions with 100 μs of pulse width and 10% of duty cycle.

The S-parameters of the fabricated SPDT T/R switches were measured with the network analyzer Rohde & Schwarz ZNB40. As shown in Figure 13a, the measured insertion loss of the T/R switch achieved 1.21 dB at 9 GHz and less than 1.36 dB in the whole X-band. The measured return loss of the proposed T/R switch was better than 10 dB in the whole X band, which corresponded well with the simulation results. The isolation of the switch was better than 34 dB within the whole X-band. Moreover, SPDT switch version-A demonstrates exceptionally high linearity up to 46 dBm, which indicates it can be employed in the Tx path with a high output power as shown in Figure 13b.

**Figure 14:**
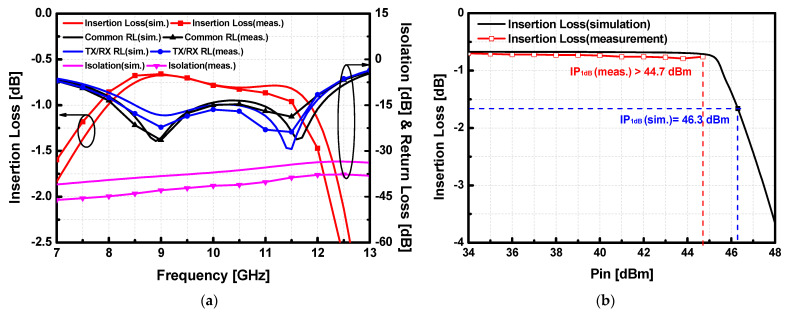
Simulated and measured (**a**) small-signal performance and (**b**) large-signal performance at 9 GHz results of the proposed SPDT T/R switch version-B (SW-B) under RF pulsed conditions with 100 μs of pulse width and 10% of duty cycle.

As shown in Figure 14a, the measured insertion loss of SW-B achieved 0.66 dB at 9 GHz and less than 1.5 dB in the whole X-band. Even though the trend of S-parameters was slightly shifted to the low frequency due to the capacitive loading effect of the shunt transistors, the insertion loss of SW-B was better than SW-A as additional lossy inductors were eliminated. The measured SW-B achieved the insertion loss below 1 dB (0.66 dB at 9 GHz) within 3.7 GHz (7.8–11.2 GHz). The improvement of the insertion loss demonstrates that the 500 Ω resistor at the gate control path does not affect the performance of the switch from the gate voltage drop. The measured return loss of the proposed SW-B was better than 10 dB in the whole X band, which corresponded well with the simulation results. The isolation of the switch was better than 35 dB within the whole X-band. SPDT T/R switch version-B demonstrates comparably high linearity with the IP_1dB_ larger than 44.7 dBm at 9 GHz as shown in Figure 14b.

Table 1 shows the performance comparison of X-band GaN SPDT switches recently reported in the literature. Both proposed SPDT T/R switches (SW-A and SW-B) have achieved outstanding performances with the lowest insertion loss and exhibited superior linearity in the X-band. Particularly, the insertion loss of both SPDT SW-A and SW-B was degraded by less than 0.1 dB up to 46 dBm and 44.7 dBm for SW-A and SW-B, respectively.

### 3.2. Driving Amplifier

**Figure 15:**
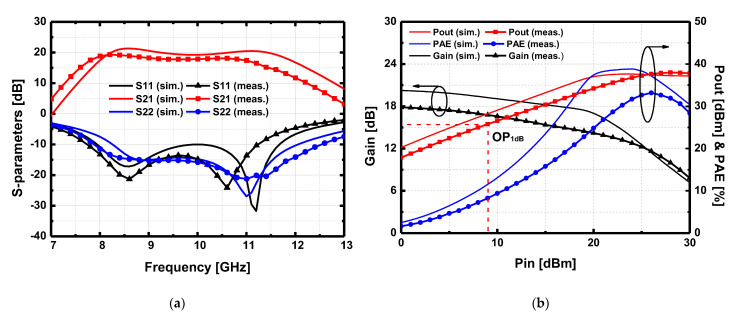
Simulated and measured (**a**) S-parameters result and (**b**) large-signal performance at 9 GHz of the driving amplifier under RF pulsed conditions with 100 μs of pulse width and 10% of duty cycle.

Figure 15a presents the measured small-signal S-parameters and pulsed large-signal performance of the proposed DA. The implemented driving amplifier was biased at *V*_GG_ = −2.2 V with *V*_DD_ = 26 V with the quiescent drain current of 130 mA. In this condition, each HEMT operates with a current density of 72 mA/mm. The measured 3 dB bandwidth was 3.5 GHz over 7.7–11.2 GHz with a peak gain of 19.3 dB at 8.2 GHz. The input and output return loss was also better than 10 dB over the 3 dB bandwidth. The measured large-signal performance result is given in Figure 15b. The measured saturated output power (*P*_sat_) achieved 38.0 dBm, and OP_1dB_ was 25.8 dBm at 9 GHz. The implemented DA can drive the HPA with the output power of 32 dBm with a large signal gain of 14 dB, which is sufficient to drive the designed HPA to the optimal saturation point.

### 3.3. High Power Amplifier

**Figure 16:**
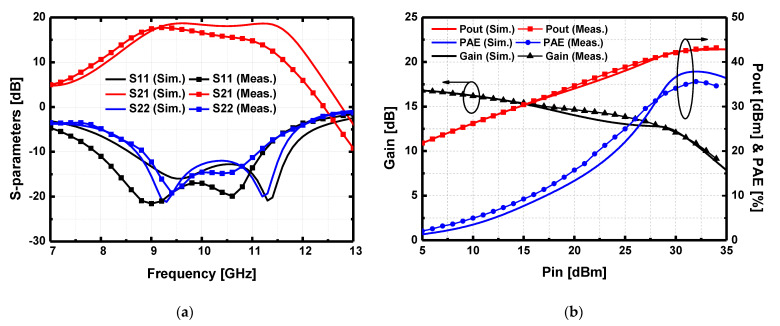
Simulated and measured (**a**) S-parameters result and (**b**) large-signal performance at 9 GHz of the implemented high power amplifier (HPA) under RF pulsed conditions with 100 μs of pulse width and 10% of duty cycle.

The implemented GaN HPA was measured for the small signal S-parameters and large-signal performance. The HPA was biased at *V*_GG_ = −2.3 V with *V*_DD_ = 26 V with the current density of each HEMT equal to 44 mA/mm. With this bias condition, the total quiescent drain current was 280 mA. Figure 16a shows the measured S-parameters, and the peak gain was 17.7 dB at 9.2 GHz, and the 3-dB bandwidth was 2.5 GHz from 8.5 to 11 GHz. Figure 16b illustrates the large-signal performance of the HPA at 9 GHz. For the large-signal measurement, the pulse-modulated RF signal was modulated with a 10% duty cycle and 100 μs pulse. The saturated output power was measured to be *P*_sat_ = 20 Watt with a large signal power gain of 10.9 dB which corresponded well with the simulation.

**Figure 17:**
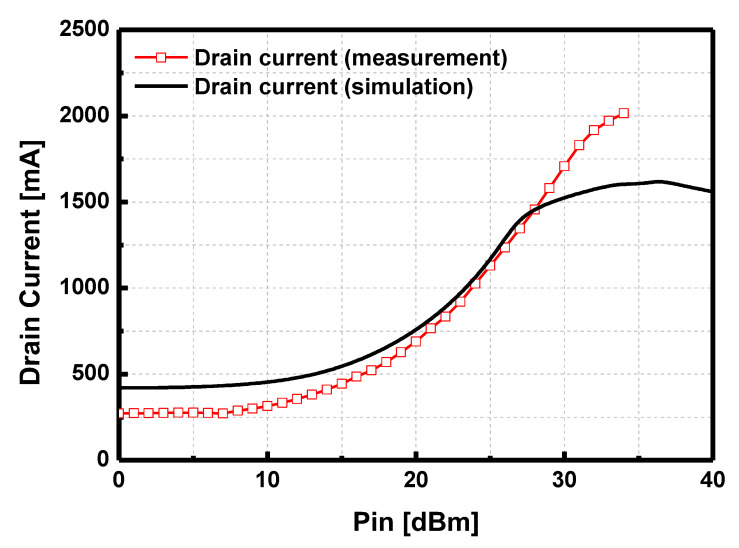
Simulated and measured drain current versus input power at 9 GHz.

At the saturation point of the output power, the peak drain current was 1.9 A, corresponding to the peak PAE of 35.6%. The drain current versus input power is compared in Figure 17.

The measured drain current was not saturated, while the simulated drain current was saturated at the output power saturation point. The discrepancy of the drain current between the simulation and measurement mainly resulted in the degradation of the peak PAE compared with that in the simulation.

Table 2 compares the implemented GaN HPA with the recently reported works in 0.25 μm at the X-band. The saturated power of the designed HPA was 43 dBm with a supply voltage of 26 V. It is noteworthy that HPA and DA were designed separately considering the design flexibility and the cost-effectiveness of the implemented transceiver front-end.

### 3.4. Low Noise Amplifier

**Figure 18:**
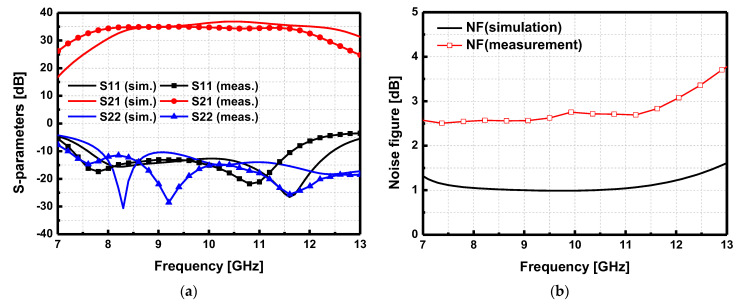
Simulated and measured (**a**) S-parameters and (**b**) noise figure of the low noise amplifier.

The implemented LNA was measured to evaluate the noise figure (NF) and S-parameters. The gate bias voltage was *V*_GG_ = −2.1 V, and the drain supply voltage was *V*_DD_ = 10 V. With this condition, the four-stage LNA consumes 1.0 watts. Figure 18a shows the comparison between the simulated and measured results of the S-parameters of the LNA. The 3 dB bandwidth was 4.4 GHz (7.6–12 GHz) with a peak gain is 34.9 dB at 9 GHz. The input and output return loss was better than 10 dB. The NF of the LNA was measured with the Y-factor method. Figure 18b presents the simulated and measured NF, which shows a large discrepancy between them. The measured NF at 9 GHz was 2.56 dB, and the maximum NF over the 3 dB bandwidth was 3 dB. It is noted that there is a large NF discrepancy between the simulation and measurement. The root cause of this discrepancy is an inaccurate device noise model provided by the PDK.

**Figure 19:**
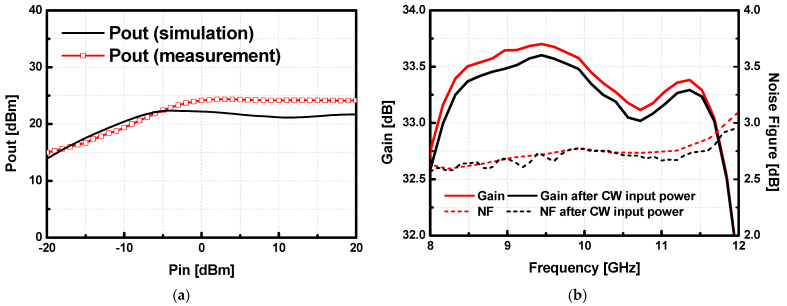
(**a**) Simulated and measured large-signal performance at 9 GHz of the low noise amplifier (LNA) under RF pulsed conditions with 100 μs of pulse width and 10% of duty cycle, and (**b**) the S-parameter and noise figure (NF) comparison before and after applying the input power of 38 dBm (100 μs of pulse width and 10% of duty-cycle) to the implemented LNA several times at X-band.

A large signal measurement was also conducted to check the output power of the LNA. Figure 19a shows the output power of the LNA at 9 GHz, the saturated output power is 24.5 dBm and a 1-dB compressed input power (IP_1dB_) is −18 dBm. Due to the drain limiter at the final stage of the LNA, output power does not exceed 25 dBm, as it is expected in the simulation. To verify the robustness of the implemented LNA to the large input power, S-parameters of the LNA were measured before and after applying the input power of 6.3 watts (100 μs of the pulse width and 10% of the duty-cycle) to the implemented LNA several times at X-band as shown in Figure 19b. There was no noticeable degradation observed after applying the high input power to the LNA. The proposed LNA is compared with other reported X-band GaN LNAs as shown in Table 3. Since the proposed LNA is required to achieve a more than 30 dB gain, four cascaded stages consume a relatively large power consumption (1.0 watts). Considering its high gain performance, the power dissipation is acceptable.

## 4. Conclusions

This paper reported the X-band GaN front-end core MMIC blocks for a low-cost transmit-receive module (TRM) for an X-band AESA radar system. All the core MMICs were implemented in 0.25 μm GaN technology including SPDT T/R switch, low noise amplifier (LNA), driving amplifier (DA), and high power amplifier (HPA). The designed SPDT T/R switches utilized shunt switches and λ/4 transmission lines (T-lines) without any series switches to substitute a bulky circulator and external limiters in a conventional TRM. The implemented SPDT T/R switch version-A (SW-A) exhibited a low insertion loss between 1.06 and 1.36 dB in the whole X-band, and IP_1dB_ higher than 46 dBm, better than 34 dB of isolation was observed. The SPDT T/R switch version-B (SW-B) achieved a lower insertion loss below 1 dB (0.66 dB at 9 GHz), better than 35 dB of isolation in 7.8–11.5 GHz, and IP_1dB_ higher than 44.7 dBm. With the proposed low-loss T/R switches, the signal-to-noise ratio (SNR) of the GaN-based TRM can be comparable to the TRM with a GaAs Rx chain with a bulky circulator and limiter. For the Tx chain, DA and HPA were co-designed to achieve the output power of 43.0 dBm. The designed HPA achieved a peak small-signal gain of 17.7 dB at 9.2 GHz and a 3 dB bandwidth of 8.5–11 GHz. The *P*_sat_ was 43 dBm at 9 GHz with a peak PAE of 35.6% and a large signal power gain of 10.9 dB. For the Rx chain, the LNA has a small-signal gain of 34.9 dB and a noise figure of 2.56 dB at 9 GHz with a wide 3 dB bandwidth covering 7.6–12 GHz. With the high feedback resistor at the gate and careful selection of the HEMT size, the implemented LNA successfully demonstrated its robustness from a high input power larger than 38 dBm at X-band.

## Figures and Tables

**Table 1:** Performance comparison of X-band GaN SPDT switches.

Ref.	This-A	This-B	[13]	[14]	[15]	[16]	[17]	[18]	[19]
Process	0.25 μm	0.25 μm	0.25 μm	0.15 μm	0.25 μm	0.8 μm	0.25 μm	0.25 μm	0.25 μm
Frequency (GHz)	8–12	8–12	0–18	0–14	8.4–10.6	8–12	2–18	8–12	8–12
Insertion Loss (dB)	1.06–1.36	0.66–1.5	0.3–1.5	<1.2	<1.1	<1.4	<2.0	1	3.5
Isolation (dB)	<34	<35	<25	>30	>28	22.2	>33	>37	35
IP_1dB_ (dBm)	>46 (IP_0.1dB_)	>44.7 (IP_0.1dB_)	>41.5	40 (IP_0.1dB_)	>42	38	>33 (IP_0.2dB_)	>39	44

**Table 2:** Performance comparison of X-band GaN high power amplifiers (HPAs).

Ref.	This	[15]	[21]	[22]	[23]	[24]	[25]	[26]	[27]
Process	0.25 μm	0.25 μm	0.25 μm	0.25 μm	0.25 μm	0.25 μm	0.25 μm	0.5 μm	0.25 μm
Frequency (GHz)	8.5–11	8.8–10.2	8–12	8.8–10.8	8.5–10.5	8–12	8.8–10.4	8.6–10.6	9–11
Small-signal Gain (dB)	17.7(34 *)	>24	24.5–28	24–26	>20	>25	25	19	N/A
*P*_sat_ (dBm)	43.0	>44.5	46.0	45.0–46.0	43.2–44.7	47.5–48.7	>41.4	42.0	45.4–46.3
PAE (%)	35.6(33.4 *)	>36	44.7	38–44	35–37	40–45	>38	33	40–52
Power Gain (dB)	10.9(25.9 *)	>18	22	17.0–18.4	17.7–19.6	21.5–22.7	18	16	16.3
Pulse (us/%)	100/10	100/30	100/10	100/10	100/10	100/10	50/15	20/1	20/10
*V*_DD_ (V)	26	30	28	28	28	28	26	25	25

* Expected results for the transmitter utilizing the implemented HPA and DA.

**Table 3:** Performance comparison of X-band GaN low noise amplifiers (LNAs).

Ref.	This	[28]	[29]	[30]	[31]	[32]	[33]	[34]	[35]	[36]
Process	0.25 μm	0.25 μm	0.25 μm	0.25 μm	0.15 μm	0.25 μm	0.25 μm	0.25 μm	N/A	0.25 μm
Frequency (GHz)	7.6–12	8–11	8–11	8–10	9–11	8.5–10.5	8–12	9.7–12.9	8–11	8–12
Gain (dB)	>33	>22	22–30.8	24–27	>13.5	>25	14	20–26	21	>16
NF (dB)	2.56	<1.6	1.6–1.95	<1.3	1.4–2.2	<2.5	1.6	1.7–2.1	2.3–2.6	2.3
P_in(max)_ (W)	>6.3	5	2.5	N/A	N/A	5	4	N/A	9	10
Pulse (μs/%)	100/10	CW	N/A	N/A	N/A	N/A	N/A	N/A	N/A	5/10
P_diss_ (W)	1.0	0.5	0.6	0.9	1.2	0.5	0.21	1.4	2.0	0.3
Gain/P_diss_ (dB/W)	>33	>44	>36.6	>26.7	>11.25	>50	>66.7	>14.29	>10.5	>53.3

## Data Availability

Not applicable.

## References

[1] Rocco G., Sergio C., Walter C., Ferdinando C., Giorgio P., Alessandro S., Marco V., Manuela S., Maurizio C., Ernesto L. (2018). S-Band GaN Single-Chip Front End for Active Electronically Scanned Array with 40-W Output Power and 1.75-dB Noise Figure. IEEE Trans. Microw. Theory Tech..

[2] Fan B., Zhao X., Zhang J., Sun Y., Yang H., Guo L.J., Zhou S. (2023). Monolithically Integrating III-Nitride Quantum Structure for Full-Spectrum White LED via Bandgap Engineering Heteroepitaxial Growth. Laser Photonics Rev..

[3] Lu T., Wang C. (2022). Radiation and Annealing Effects on GaN MOSFETs Irradiated by 1 MeV Electrons. Electronics.

[4] Trew R.J. (2002). SiC and GaN transistors—Is there one winner for microwave power applications?. Proc. IEEE.

[5] Angelov I., Zirath H., Roshman N. (1992). A new empirical nonlinear model for HEMT and MESFET devices. IEEE Trans. Microw. Theory Tech..

[6] Sadi T., Schwierz F. A Continuous Physics-Based Electrothermal Compact Model for the Study of Non Linearities in III–V HEMTs. Proceedings of the 40th IEEE International Conference on Solid-State Device Research Conference (ESSDERC).

[7] Dasgupta A., Ghosh S., Chauhan Y.S., Khandelwal S. ASMHEMT: Compact model for GaN HEMTs. Proceedings of the IEEE International Conference on Electron Devices and Solid-State Circuits (EDSSC).

[8] Radhakrishna U., Imada T., Palacios T., Antoniadis D. (2014). MIT virtual source GaNFET-high voltage (MVSG-HV) model: A physics based compact model for HV-GaN HEMTs. Phys. Status Solidi C.

[9] Jazaeri F., Sallese J.-M. (2019). Charge-based EPFL HEMT model. IEEE Trans. Electron Devices.

[10] Touchaei B.J., Shalchian M. (2022). Non-Quasi-Static Intrinsic GaN-HEMT Model. IEEE Trans. Electron Devices.

[11] Nazir M.S., Kushwaha P., Pampori A., Ahsan S.A., Chauhan Y.S. (2022). Electrical Characterization and Modeling of GaN HEMTs at Cryogenic Temperatures. IEEE Trans. Electron Devices.

[12] Albahrani S.A., Mahajan D., Hodges J., Chauhan Y.S., Khandelwal S. (2019). ASM GaN: Industry Standard Model for GaN RF and Power Devices—Part-II: Modeling of Charge Trapping. IEEE Trans. Electron Devices.

[13] Campbell C.F., Dumka D.C., Kao M.-Y. Design considerations for GaN-based MMICs. Proceedings of the 2009 IEEE International Conference on Microwaves, Communications, Antennas and Electronics Systems.

[14] Osmanoglu S., Ozbay E. X-Band High Power GaN SPDT MMIC RF Switches. Proceedings of the 2019 European Microwave Conference in Central Europe (EuMCE).

[15] D’Angelo S., Biondi A., Scappaviva F., Resca D., Monaco V.A. A GaN MMIC chipset suitable for integration in future X-band spaceborne radar T/R module Frontends. Proceedings of the 2016 21st International Conference on Microwave, Radar and Wireless Communications (MIKON).

[16] Hettak K., Ross T.N., Cormier G., Wight J.S. (2013). Broadband High-Power GaN SPDT Switch Using Stacked-Shunt FETs and Resonance Inductors. Microw. Opt. Technol. Lett..

[17] Thorsell M., Fagerlind M., Andersson K., Billstrom N., Rorsman N. (2010). An X-Band AlGaN/GaN MMIC Receiver Front-End. IEEE Microw. Wirel. Compon. Lett..

[18] Ciccognani W., Ferrari M., Limiti E. (2010). High isolation microstrip GaN-HEMT Single-FET Switch. Int. J. RF Microw. Comput.-Aided Eng..

[19] Janssen J., Hilton K.P., Maclean J.O., Wallis D.J., Powell J., Uren M., Martin T., van Heijningen M., van Vliet F. X-Band GaN SPDT MMIC with over 25 Watt Linear Power Handling. Proceedings of the 2008 European Microwave Integrated Circuit Conference.

[20] Rudolph M., Behtash R., Doerner R., Hirche K., Wurfl J., Heinrich W., Trankle G. (2007). Analysis of the Survivability of GaN Low-Noise Amplifiers. IEEE Trans. Microw. Theory Tech..

[21] Chen R., Li R., Zhou S., Chen S., Huang J., Wang Z. (2019). An X-Band 40 W Power Amplifier GaN MMIC Design by Using Equivalent Output Impedance Model. Electronics.

[22] Shin D.-H., Yom I.-B., Kim D.-W. X-band GaN MMIC power amplifier for the SSPA of a SAR system. Proceedings of the 2017 IEEE International Symposium on Radio-Frequency Integration Technology (RFIT).

[23] Bae K.-T., Lee I.-J., Kang B., Sim S., Jeon L., Kim D.-W. (2017). X-Band GaN Power Amplifier MMIC with a Third Harmonic-Tuned Circuit. Electronics.

[24] Tao H.-Q., Hong W., Zhang B., Yu X.-M. (2017). A Compact 60W X-Band GaN HEMT Power Amplifier MMIC. IEEE Microw. Wirel. Compon. Lett..

[25] Resca D., Raffo A., Di Falco S., Scappaviva F., Vadalà V., Vannini G. (2014). X-Band GaN Power Amplifier for Future Generation SAR Systems. IEEE Microw. Wirel. Compon. Lett..

[26] Giofre R., Colantonio P., Giannini F. (2013). X-band MMIC GaN power amplifier for SAR systems. Microw. Opt. Technol. Lett..

[27] Piotrowicz S., Ouarch Z., Chartier E., Aubry R., Callet G., Floriot D., Jacquet J.C., Jardel O., Morvan E., Reveyrand T. 43W, 52% PAE X-Band AlGaN/GaN HEMTs MMIC amplifiers. Proceedings of the 2010 IEEE MTT-S International Microwave Symposium.

[28] Yağbasan Ç., Aktuğ A. Robust X-band GaN LNA with Integrated Active Limiter. Proceedings of the 2018 13th European Microwave Integrated Circuits Conference (EuMIC).

[29] Kazan O., Kocer F., Civi O.A. An X-Band Robust GaN Low-Noise Amplifier MMIC with sub 2 dB Noise Figure. Proceedings of the 2018 13th European Microwave Integrated Circuits Conference (EuMIC).

[30] Vittori M., Colangeli S., Ciccognani W., Salvucci A., Polli G., Limiti E. High performance X-band LNAs using a 0.25 μm GaN technology. Proceedings of the 2017 13th Conference on Ph.D. Research in Microelectronics and Electronics (PRIME).

[31] Kobayashi K.W., Campbell C., Lee C., Gallagher J., Shust J., Botelho A. A reconfigurable S-/X-band GaN cascode LNA MMIC. Proceedings of the 2017 IEEE Compound Semiconductor Integrated Circuit Symposium (CSICS).

[32] Kim B., Gao W. X-Band Robust Current-Shared GaN Low Noise Amplifier for Receiver Applications. Proceedings of the 2016 IEEE Compound Semiconductor Integrated Circuit Symposium (CSICS).

[33] Schuh P., Reber R. Robust X-band low noise limiting amplifiers. Proceedings of the 2013 IEEE MTT-S International Microwave Symposium Digest (MTT).

[34] Chang W., Jeon G.-I., Park Y.-R., Lee S., Mun J.-K. X-band low noise amplifier MMIC using AlGaN/GaN HEMT technology on SiC substrate. Proceedings of the 2013 Asia-Pacific Microwave Conference Proceedings (APMC).

[35] Bettidi A., Corsaro F., Cetronio A., Nanni A., Peroni M., Romanini P. X-Band GaN-HEMT LNA performance versus robustness trade-off. Proceedings of the 2009 European Microwave Integrated Circuits Conference (EuMIC).

[36] Janssen J.P.B., van Heijningen M., Provenzano G., Visser G.C., Morvan E., van Vliet F.E. X-Band Robust AlGaN/GaN Receiver MMICs with over 41 dBm Power Handling. Proceedings of the 2008 IEEE Compound Semiconductor Integrated Circuits Symposium.

